# Is aerobic combined with resistance training a more suitable exercise program for obese college students? Evidence-based on subjective reports and objective measurements

**DOI:** 10.3389/fphys.2025.1675205

**Published:** 2025-12-04

**Authors:** Yan Li, Yanbin Hu

**Affiliations:** 1 College of Liberal Studies (Sports Work Department), Chongqing Industry Polytechnic University, Chongqing, China; 2 Department of Physical Education Teaching, Beijing University of Chinese Medicine, Beijing, China

**Keywords:** obese college students, aerobic combined resistance training, heart rate variability, lipidmetabolism, subjective exercise experience

## Abstract

**Objective:**

This study aims to investigate the exercise programs that are more suitable for obese college students.

**Methods:**

A randomized controlled experimental design was used to randomly divide 57 obese college students into Baduanjin combined resistance training group (BRTG, n = 19), resistance training group (RTG, n = 19), and control group (CG, n = 19).

**Results:**

1) After an 8-week intervention, BMI, PBF, and VFI of BRTG and RTG significantly decreased, while MM significantly increased; Meanwhile, PBF and MM of BRTG were significantly lower than that of RTG. 2) The SDNN, RMSSD, and HFnorm of BRTG and RTG were significantly increased after intervention, and LFnorm and LF/HF of BRTG and RTG were significantly decreased; Meanwhile, LFnorm and LF/HF of BRTG were significantly lower than RTG, while HFnorm was significantly higher than RTG. 3) The TC, TG, and LDL-C of BRTG and RTG were significantly decreased, while HDL-C was significantly increased after intervention. 4) Compared with single resistance training, Baduanjin combined with resistance training intervention is more conducive to enhancing the subjective exercise experience and compliance of obese college students.

**Conclusion:**

Moderate-intensity aerobic combined with resistance training is a more suitable exercise program for obese college students and has greater application potential in health promotion.

## 1 Introduction

Obesity is a global epidemic that affects about 5.5% of college students in different geographical regions (Peltzer et al., 2014) and is regarded as one of the main problems affecting the health of college students (Woli et al., 2023; Song et al., 2024). The university stage is a crucial period for the formation of individual health behaviors. Early prevention and intervention of obesity play an important role in health in later life (García-Hermoso et al., 2020). Obesity is a pathological state in which the fat component of the body exceeds the average amount of normal people (Lüscher, 2020; Cho et al., 2022). For example, fat accumulation in the body often affects the normal metabolism of blood lipids and liver function (Ye et al., 2016; Racil et al., 2015), and blood lipid levels can often reflect the body’s lipid metabolism. In individuals with obesity, increased body fat content is associated with abnormal lipid metabolism, characterized by decreased levels of total cholesterol (TC), triglycerides (TG), low-density lipoprotein cholesterol (LDL-C), and high-density lipoprotein cholesterol (HDL-C) (Lu and Xie, 2021). Meanwhile, obese patients often have abnormal cardiovascular autonomic nervous system function, which is clinically manifested as sympathetic dominance in the sympathetic-vagal tension balance, impaired pressure-sensitive reflex function, reduced vasodilatation, resting tachycardia, and abnormal myocardial blood flow regulation, leading to impaired cardiac function (Lefrandt et al., 2010). It can be seen that obesity is an important threat to the healthy growth of college students. How to effectively regulate the body function targets related to obesity is crucial to improving the health of obese college students.

Exercise is recognized as an effective way to improve individual obesity (Slentz et al., 2004; Catenacci et al., 2019), which can effectively reduce the weight of obese college students and reduce the health risks of obesity (Kim et al., 2018). The main mechanism of exercise intervention in obesity is to reduce body fat by increasing energy consumption, promoting energy metabolism and the production of anti-inflammatory cytokines, and stabilizing the concentrations of lipids, blood sugar, and hormones (Weng and Schuppan, 2013). Currently, the effects and mechanisms of different types of exercise on health promotion for obese people have received widespread attention. For example, Jin and Zhu (2017) conducted a 6-week aerobic exercise intervention on obese college students and found that the subjects’ body shape and blood lipid metabolism levels improved after the intervention. Aerobic exercise can not only significantly improve the body composition, such as BMI and percent of body fat, of obese college students (Qi et al., 2020) but also effectively improve cardiovascular autonomic nervous system dysfunction in obese college students (Voulgari et al., 2013). Meanwhile, resistance training can enhance basal metabolic rate and improve lipid oxidation in adolescents with obesity, thereby reducing body fat (Monteiro et al., 2015), and it also contributes to decreased high-density lipoprotein (HDL) levels (Williams et al., 2011) and attenuated sympathetic nervous activity (Anunciao et al., 2016). As the research deepens, researchers have realized that the weight loss benefits of single exercise intervention have limitations. For example, long-term aerobic exercise alone has shortcomings such as long exercise time and monotonous exercise process, which leads to a decrease in exercise enthusiasm and effect (Fang et al., 2020), so the intervention model of aerobics combined with resistance has gradually become popular (Ho et al., 2012; Chen et al., 2019). Rustaden et al. (2018) showed that aerobic exercise can achieve weight loss by reducing body fat mass, while resistance training can increase resting metabolic rate by increasing muscle mass (MM), thereby achieving the purpose of fat loss. Further research found that an exercise program combining aerobic training with resistance training may be more effective in improving body fat content, and insulin sensitivity is greater after exercise intervention (Poehlman et al., 2000; Suh et al., 2011). Qi et al. (2020) also found that compared with simple aerobic exercise, aerobic exercise combined with resistance exercise has a more significant effect in reducing abdominal fat, and visceral fat and improving cardiopulmonary function in obese college students. Furthermore, the lack of interest in exercise is one of the main factors leading to the decline in physical fitness among adolescent students (Zhang, 2009). During the exercise process, a good sense of experience will help enhance an individual’s exercise intention and persistence; otherwise, it will inhibit the exercise intention, increase fatigue, restrict the implementation of the plan, and lead to withdrawal tendencies (Bryan et al., 2007; Zhang and Dong, 2017). Among them, compared with resistance training, aerobic combined with resistance training has a more significant effect on the aerobic capacity and muscle fatigue resistance of adults (Calders et al., 2012), and it has a better effect on improving the fatigue perception and exercise tolerance of patients with multiple sclerosis (Grazioli et al., 2019). From this, it can be inferred that aerobics combined with resistance training may have greater application potential for obese people.

In China, the rate of overweight or obesity among college students has been on the rise over the past 30 years (Wang and Wang, 2018), with the overweight and obesity rates of residents aged 18 and above being 34.3% and 16.4%, respectively, an increase of 4.2% and 4.5% from 2015 (Disease Preventi on and Control Bureau of the PRC, 2022). The Baduanjin is one of the traditional Chinese aerobic exercise programs, it is essentially a traditional exercise therapy with the characteristics of convenient exercise, simple movements, and suitable for all ages (Xiao et al., 2023). Moreover, the exercise intensity of Baduanjin is moderate, the exercise risk is relatively low, and the compliance is relatively high (Zheng et al., 2024). A study has shown that long-term Baduanjin practice can reduce blood sugar in obese female diabetic patients and has a certain improvement effect on some obesity and blood lipid indicators in the body (Liu et al., 2018). Baduanjin has a good therapeutic effect on coronary heart disease, essential hypertension, chronic heart failure, arrhythmia, and cardiovascular-related risk factors (Wang and Mo, 2019). However, previous studies have seldom examined the health promotion effects of combining Baduanjin with resistance training on obese college students, nor have they adequately integrated subjective perceptions (such as exercise experience) with objective measurements (such as heart rate variability and lipid metabolism) for a comparative analysis. Based on this, the present study aims to investigate the effects of single resistance training and Baduanjin combined with resistance training on both subjective experiences and objective measurements among obese college students, compare the differences in effectiveness among various intervention methods, and subsequently summarize an effective exercise regimen suitable for this population. Meanwhile, the hypotheses of this study are as follows: H1: Both single resistance training and Baduanjin combined with resistance training will have positive health promotion effects on obese college students; H2: The combined aerobic and resistance training will yield greater positive benefits in terms of both subjective exercise experience and objective measurements for participants.

## 2 Participants and methods

### 2.1 Participants

This study employed a randomized controlled trial design. A total of 60 obese college students were voluntarily recruited from Chongqing Industry Polytechnic College, China, with 57 participants (41 boys and 16 girls) meeting the predefined inclusion/exclusion criteria and finally enrolled in the experimental trial. The average age of boys was 19.67 ± 1.02 years, the average body height was 1.71 ± 0.58 m, and the average body weight was 86.22 ± 10.39 kg. The average age of girls was 19.81 ± 1.05 years, the average body height was 1.60 ± 0.52 m, and the average body weight was 73.92 ± 8.87 kg. This study recruited participants voluntarily from February to March 2024, followed by an intervention period lasting from March to June 2024.

Inclusion criteria: 1) the participants were 18–22 years old; 2) full-time college students; 3) voluntary participants; 4) no regular physical exercise habits; 5) no physical disabilities or medical problems that prevented or prohibited them from participating in moderate-intensity aerobic exercise as assessed by the Physical Activity Readiness Questionnaire (PAR-Q); 6) met the obesity standards in the National Student Physical Fitness Standards (2014 Edition), BMI ≥28 kg/m^2^ (Ministry of Education of the People’s Republic of China, 2020; Zhao and Liang, 2019). Exclusion criteria: 1) history of heart disease; 2) history of mental illness; 3) those who have regular exercise habits or have received exercise intervention in the past 3 months. This study was approved by the Ethics Committee of the School of Physical Education, Southwest University (SWU-TY202105), followed the Declaration of Helsinki, and obtained written informed consent from all participants.

### 2.2 Methods

#### 2.2.1 Procedure

**FIGURE 1:**
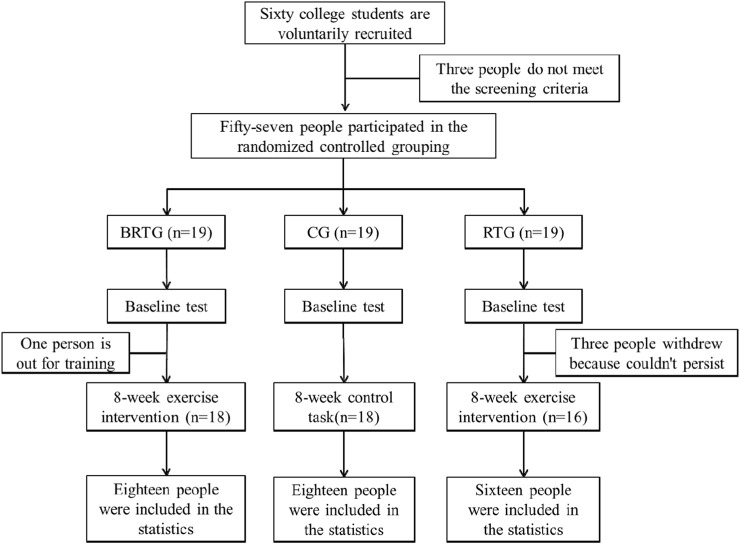
CONSORT flow diagram.

All 57 participants who have been screened are required to complete the following items (please refer to Figure 1 for the CONSORT flow diagram).

1. Baseline test: All participants were required to undergo the same baseline test before entering the formal experiment, including demographic information, body composition measurement, autonomic nervous system activity, and adipose cytokine collection.
2. Experimental grouping and intervention: After the baseline test, a double-blind randomization method using random numbers was applied to all participants (both the researchers and participants were unaware of the group assignments in advance). The participants were divided into three groups: the Baduanjin combined with resistance training group (BRTG, n = 19), the resistance training group (RTG, n = 19), and the control group (CG, n = 19). The intervention in this study was administered three times per week, with each session lasting 40 min, over a total duration of 8 weeks. The overall exercise intensity was moderate (55%–70% of HR_max_, where HR_max_ = 206.9–0.67 × age), and the heart rate during exercise was monitored using a Polar RCX3 heart rate monitor. The specific intervention time was Tuesday, Thursday, and Saturday every week, and the intervention location was the indoor or outdoor sports field of Chongqing Industry Polytechnic College in Chongqing. It should be noted that throughout the intervention period, all participants were instructed to avoid high-fat and high-calorie diets and, unless under special circumstances, were required to dine uniformly in the campus cafeteria. Additionally, they were also advised to refrain from engaging in any additional moderate-to-high intensity physical activities.

Among them, BRTG received Baduanjin combined with resistance training intervention, specifically, the first 3 min of warm-up activities to achieve the effect of warm-up, then about 15 min of Baduanjin exercise, then about 18 min of resistance training with an interval of 1 min, and finally 3 min of stretching and relaxation. RTG received resistance training intervention, specifically, they first performed 3 min of warm-up activities to achieve a warm-up effect and then performed the same resistance training as BRTG. It should be pointed out that to ensure that the duration of a single exercise intervention in the two experimental groups is consistent as much as possible, RTG needs to repeat the main movements of the resistance training twice, and finally perform 3 min of stretching and relaxation. CG did not receive any intervention and only carried out daily college life.

3. Post-test: All participants were required to undergo the same index test as the baseline test after completing the 8-week intervention. It should be noted that during the experimental intervention process, two subjects (one for BRTG and one for CG) could not stay at school for a long time due to the need for external training, and three withdrew because they could not persist in exercising (all RTG). Therefore, a total of 52 samples were ultimately included in the data analysis (18 for BRTG, 16 for RTG, and 18 for CG). A *post hoc* power analysis using G*Power software revealed that with an effect size of 0.25 and α set at 0.05, the achieved statistical power reached 0.89 with the current sample size, which met the study requirements.

#### 2.2.2 Intervention content

##### 2.2.2.1 Baduanjin

The content is taught and supervised by professional Baduanjin teachers. Refer to the “Health Qigong Baduanjin” specification (Health Qigong, 2003), the specific movements include preparatory posture, two hands to the sky to regulate the three energizers, left and right bows like shooting eagles, regulating the spleen and stomach with one arm, looking back for five kinds of labor and seven kinds of injuries, shaking the head and tail to relieve heart fire, two hands climbing the feet to strengthen the kidneys and waist, clenching fists and glaring to increase strength, shaking the back seven times to eliminate all diseases and closing posture, a total of 10 movements. The exercise intensity of Baduanjin exercise in this study is moderate (55% HR_max_ to 70% HR_max_).

##### 2.2.2.2 Resistance training

The content was compiled and supervised by professional fitness coaches, including flexion and extension exercises of the shoulder joint, elbow joint, hip joint, knee joint, and trunk muscle groups, such as push-ups, single-leg lunges, high pull-downs, supine leg raises, seated leg presses, etc. The exercise intensity was about 65%–70% of the maximum repetition load (one repetition maximum, 1RM), three groups were performed each time, with a rest period of 1min between groups. Each group of movements included six exercise links, and each link was repeated 6–8 times. To ensure the safety and effectiveness of the exercise program, the training process was supervised by medical staff, and all participants did not participate in other forms of regular resistance exercise during the experiment.

#### 2.2.3 Measurement tools

The outcomes of this study consisted of both subjective reports and objective measurements. The primary outcomes included subjective exercise experience, heart rate variability, and changes in lipid metabolism levels, while the secondary outcome was body composition. The data of participants at different stages were collected and evaluated by dedicated research staff.

##### 2.2.3.1 The subjective exercise experience scale (SEES)

The Subjective Exercise Experience Scale of Mcauley and Courneya, (1994) was adopted to evaluate the subjective experience of the participants during the exercise process. This scale includes three dimensions: positive wellbeing (4 items), psychological distress (4 items), and fatigue (4 items), with a total of 12 items. The Likert 7-level scoring method is adopted, among them, psychological distress and fatigue are scored in reverse. The total score of SEES is obtained by adding the three dimensions, and the score range is 12–84 points, and the higher the score, the better the subjective exercise experience. In this study, the factor loadings of SEES were greater than 0.50, the AVE exceeded 0.60, and the composite reliability (CR) was above 0.70. Furthermore, internal consistency tests revealed that the Cronbach’s α coefficients of the scale was 0.82 and 0.85 before and after the intervention, respectively, demonstrating good validity and reliability.

##### 2.2.3.2 Body composition measurement

The body composition indicators of participants were measured using a body composition analyzer (InBody 270, Republic of Korea), including body mass index (BMI), percent of body fat (PBF), visceral fat index (visceral fat index, VFI, normal value is 1–9), and muscle mass (MM, kg). During the testing procedure, participants were required to maintain an upright posture, stand barefoot on the designated electrode positions of the instrument, and grip the electrode areas on the handles with both hands while avoiding body movement. Each individual test lasted approximately 50 s. Throughout this process, participants’ body composition indicators were automatically collected, and upon completion of the test, the corresponding data were transmitted to a computer.

##### 2.2.3.3 Heart rate variability measurement

The heart rate variability (HRV) monitor (HeaLink-R211B Micro-ECG, China) was used to monitor the cardiac autonomic nerves. V4R and V5 dual leads were used, and disposable ECG electrodes made of Ag/AgCl were attached to the participants’ right clavicle midline and the lowest end of the left ribs. The bandwidth of the device was 0.5–40 Hz, and the sampling frequency was 400 Hz. The acquisition indicators consist of time domain indicators and frequency domain indicators, the former including the standard deviation of continuous normal R-R intervals (SDNN) and the root mean square value of the difference between adjacent R-R intervals (RMSSD); the latter including standardized low-frequency power (LFnorm), standardized high-frequency power (HFnorm), and low-high frequency ratio (LF/HF). During the test, the subjects adjusted their breathing and sat quietly on the chair, closed their eyes, and kept silent, and the surrounding environment was kept as quiet as possible. The single test lasted for 5 min (Malik and Camm, 1995).

##### 2.2.3.4 Lipid metabolism indicators

Lipid metabolism indices were determined by enzyme colorimetry. Fasting peripheral venous blood samples (2 mL) were collected from all participants in the morning (8–9 a.m.) before and after the intervention. The samples were left to stand at room temperature for 30 min, then centrifuged at 4,000 r/min for 10 min, after which the serum was extracted. The blood lipid analysis was conducted using the 7,600 fully automatic biochemical analyzer (Hitachi Corporation, Japan), and the detection indicators included total cholesterol (TC), triglyceride (TG), low-density lipoprotein cholesterol (LDL-C), and high-density lipoprotein cholesterol (HDL-C) were determined using a fully automatic biochemical analyzer.

### 2.3 Data

SPSS 26.0 software was used for statistics and analysis of data. Before conducting the formal analysis, the Shapiro-Wilk test was used to examine the distribution of the indicators, revealing that the data in all groups followed a normal distribution (p > 0.05). Since this study only collected data at two time points (baseline and week 8), using repeated measures ANOVA might increase statistical bias. Therefore, independent samples t-tests were first employed for descriptive analysis of the baseline characteristics. Subsequently, one-way ANOVA combined with paired samples t-tests was employed to examine between-group and within-group differences across various indicators (subjective exercise experience, body composition, heart rate variability, and lipid metabolism) at different time points (baseline, week 8) and among different groups (BRTG, RTG, and CG). During the analysis, the confidence interval was set at 95% CI with 5,000 bootstrap samples, and the between-group effect size was reported using η^2^. When the sphericity assumption was violated, the degrees of freedom and p-values were adjusted using the Greenhouse-Geisser correction, and *post hoc* comparisons were conducted with the Bonferroni method. The significance level of statistical tests was set at p < 0.05.

## 3 Results

### 3.1 The variation characteristics of subjective exercise experience levels before and after exercise intervention

**FIGURE 2:**
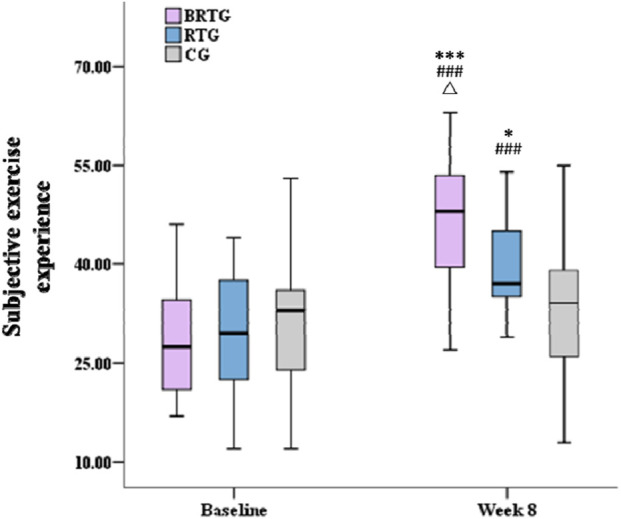
The differences in the subjective exercise experience of the participants before and after the exercise intervention. Note: *p < 0.05, ***p < 0.001, compared with CG; ###p < 0.001, compared with the baseline, △p < 0.05, compared with RTG.

There was no significant intergroup difference in the total score of subjective exercise experience among the three groups at baseline (F = 0.24, p > 0.05, η^2^ = 0.01), while there was a significant difference among the three groups after 8 weeks of intervention (F = 8.92, p < 0.01, η^2^ = 0.28). Among them, both BRTG (p < 0.001) and RTG (p < 0.05) were significantly higher than CG, and BRTG was significantly higher than RTG (p < 0.05). Within-group comparisons revealed that after the 8-week intervention, the subjective exercise experiences of BRTG (t = −7.03, p < 0.001) and RTG (t = −6.47, p < 0.001) were significantly higher than their respective baseline levels, while there was no significant change in CG (p > 0.05) (see Figure 2).

### 3.2 The variation characteristics of body composition before and after exercise intervention

At baseline, no significant between-group differences were observed in BMI (F = 0.89, p > 0.05, η^2^ = 0.04), BFR (F = 0.69, p > 0.05, η^2^ = 0.03), VFI (F = 0.47, p > 0.05, η^2^ = 0.02), and MM (F = 0.39, p > 0.05, η^2^ = 0.02) among the three groups, respectively. However, after the 8-week intervention, significant between-group differences emerged in BMI (F = 4.68, p < 0.05, η^2^ = 0.17), BFR (F = 14.28, p < 0.001, η^2^ = 0.38), VFI (F = 4.84, p < 0.05, η^2^ = 0.17), and MM (F = 8.81, p < 0.01, η^2^ = 0.28), respectively. Among them, after 8 weeks of intervention, the BMI (p < 0.01, p < 0.05, respectively), PBF (p < 0.001, p < 0.01, respectively), and VFI (p < 0.01, p < 0.05, respectively) of BRTG and RTG showed a downward trend and were significantly lower than those of CG. However, the MM of BRTG and RTG both showed an upward trend and was significantly higher than CG (p < 0.05, p < 0.001, respectively). It should be pointed out that after 8 weeks of intervention, the PBF of BRTG was significantly lower than that of RTG (p < 0.05), while the MM of RTG was significantly higher than that of BRTG (p < 0.05) (see Table 1).

**TABLE 1:** Effects of an exercise intervention on body composition of participants.

Variable	Testing period	BRTG (a)	RTG (b)	CG (c)	F	Compare (among groups)
BMI	Baseline	29.44 ± 0.84	29.39 ± 1.13	29.04 ± 0.81	0.89	——
	Week 8	28.24 ± 0.97	28.36 ± 1.19	29.25 ± 0.95	4.68*	a<c**, b < c*
	t	8.60***	7.38***	−1.36		
PBF	Baseline	37.46 ± 1.10	37.76 ± 0.97	37.33 ± 1.13	0.69	——
	Week 8	35.33 ± 1.33	36.30 ± 0.93	37.46 ± 1.11	14.28***	a<c***,b < c**,a<b*
	t	12.95***	6.92***	−1.04		
VFI	Baseline	12.98 ± 0.71	13.06 ± 0.82	13.22 ± 0.73	0.47	——
	Week 8	12.31 ± 0.80	12.46 ± 0.85	13.14 ± 0.78	4.84*	a<c**, b < c*
	t	6.99***	8.16***	1.27		
MM	Baseline	45.33 ± 2.67	45.64 ± 2.00	44.94 ± 2.09	0.39	——
	Week 8	46.59 ± 1.64	48.07 ± 2.24	45.08 ± 2.19	8.81**	a>c*,b > c***,b>a*
	t	−3.57**	−4.20**	−1.66		

* indicates p < 0.05, ** indicates p < 0.01, *** indicates p < 0.001.

### 3.3 The variation characteristics of HRV before and after exercise intervention

At baseline, no significant between-group differences were observed in SDNN (F = 0.17, p > 0.05, η^2^ = 0.01), RMSSD (F = 0.16, p > 0.05, η^2^ = 0.01), LFnorm (F = 0.13, p > 0.05, η^2^ = 0.01), HFnorm (F = 0.26, p > 0.05, η^2^ = 0.01), and LF/HF (F = 0.23, p > 0.05, η^2^ = 0.01) among the three groups, respectively. However, after the 8-week intervention, significant between-group differences emerged in SDNN (F = 3.67, p < 0.05, η^2^ = 0.14), RMSSD (F = 3.76, p < 0.05, η^2^ = 0.14), LFnorm (F = 9.37, p < 0.001, η^2^ = 0.29), HFnorm (F = 8.82, p < 0.01, η^2^ = 0.26), and LF/HF (F = 14.99, p < 0.001, η^2^ = 0.39), respectively. On the one hand, the SDNN (p < 0.05, p < 0.05, respectively) and RMSSD (p < 0.05, p < 0.05, respectively) of BRTG and RTG both showed an upward trend and were significantly higher than CG after the intervention. On the other hand, the LFnorm (p < 0.001, p < 0.05, respectively) and LF/HF (p < 0.001, p < 0.01, respectively) of BRTG and RTG both showed a downward trend and were significantly lower than CG after the intervention, while the HFnorm (p < 0.001, p < 0.05, respectively) showed an upward trend and was significantly higher than CG. Furthermore, the LFnorm (p < 0.05) and LF/HF (p < 0.05) of BRTG were also significantly lower than those of RTG after 8 weeks of intervention, and the HFnorm was significantly higher than that of BRTG (p < 0.05) (see Table 2).

**TABLE 2:** Effects of exercise intervention on HRV of participantiks.

Variable	Testing period	BRTG (a)	RTG (b)	CG (c)	F	Compare (among groups)
SDNN (ms)	Baseline	115.35 ± 16.60	118.46 ± 15.92	116.54 ± 13.27	0.17	——
	Week 8	132.54 ± 19.62	130.69 ± 15.63	117.75 ± 16.01	3.67*	a>c*, b > c*
	t	−4.49***	−5.69***	−0.56		
RMSSD (ms)	Baseline	26.65 ± 10.75	27.63 ± 9.43	25.68 ± 9.62	0.16	——
	Week 8	34.98 ± 10.09	33.78 ± 10.22	26.36 ± 9.14	3.76*	a>c*, b > c*
	t	−8.34***	−5.20***	−0.88		
LFnorm (%)	Baseline	67.44 ± 7.14	66.94 ± 6.32	67.03 ± 6.56	0.13	——
	Week 8	60.25 ± 6.08	63.26 ± 6.64	68.41 ± 6.53	9.37***	a<c***,b < c*,a<b*
	t	8.54***	2.64*	−0.93		
HFnorm (%)	Baseline	32.55 ± 5.70	33.06 ± 5.56	32.96 ± 5.44	0.26	——
	Week 8	39.74 ± 5.39	36.73 ± 5.21	31.58 ± 4.98	8.82**	a>c***,b > c*,a>b*
	t	−3.67**	−2.35*	0.31		
LF/HF	Baseline	2.69 ± 0.76	2.55 ± 0.54	2.59 ± 0.55	0.23	——
	Week 8	1.75 ± 0.42	2.14 ± 0.52	2.62 ± 0.44	14.99***	a<c***,b < c**,a<b*
	t	8.17***	2.51*	−0.42		

* indicates p < 0.05, ** indicates p < 0.01, *** indicates p < 0.001.

### 3.4 The variation characteristics of lipid metabolism before and after exercise intervention

At baseline, no significant between-group differences were observed in TC (F = 0.28, p > 0.05, η^2^ = 0.01), TG (F = 0.14, p > 0.05, η^2^ = 0.01), LDL-C (F = 0.21, p > 0.05, η^2^ = 0.01), and HDL-C (F = 0.08, p > 0.05, η^2^ = 0.01) among the three groups, respectively. However, after the 8-week intervention, significant between-group differences emerged in TC (F = 4.28, p < 0.05, η^2^ = 0.16), TG (F = 8.04, p < 0.01, η^2^ = 0.26), LDL-C (F = 4.40, p < 0.05, η^2^ = 0.16), and HDL-C (F = 4.90, p < 0.05, η^2^ = 0.18), respectively. Among them, after 8 weeks of intervention, the TC (p < 0.01, p < 0.05, respectively), TG (p < 0.01, p < 0.01, respectively), and LDL-C (p < 0.05, p < 0.05, respectively) in BRTG and RTG showed a downward trend and were significantly lower than CG. However, HDL-C showed an upward trend and was significantly higher than CG (p < 0.01, p < 0.05, respectively), while there was no significant change in CG before and after the intervention (see Table 3).

**TABLE 3:** Effects of exercise intervention on lipid metabolism of participants.

Variable	Testing period	BRTG (a)	RTG (b)	CG (c)	F	Compare (among groups)
TC	Baseline	4.49 ± 0.42	4.61 ± 0.40	4.57 ± 0.49	0.28	——
	Week 8	4.22 ± 0.40	4.33 ± 0.35	4.62 ± 0.47	4.28*	a<c**, b < c*
	t	9.30***	6.26***	−1.32		
TG	Baseline	1.72 ± 0.25	1.76 ± 0.17	1.74 ± 0.19	0.14	——
	Week 8	1.52 ± 0.24	1.56 ± 0.22	1.80 ± 0.20	8.04**	a<c**,b < c**
	t	5.86***	4.39**	−2.05		
LDL-C	Baseline	2.68 ± 0.23	2.64 ± 0.29	2.70 ± 0.33	0.21	——
	Week 8	2.41 ± 0.33	2.43 ± 0.40	2.72 ± 0.28	4.40*	a<c*,b < c*
	t	5.75***	2.24*	−0.20		
HDL-C	Baseline	1.33 ± 1.75	1.31 ± 0.22	1.34 ± 0.23	0.08	——
	Week 8	1.58 ± 0.27	1.54 ± 0.23	1.33 ± 0.25	4.90*	a>c**,b > c*
	t	−6.23***	−3.32**	0.39		

* indicates p < 0.05, ** indicates p < 0.01, *** indicates p < 0.001.

## 4 Discussion

This study clarified the differences in the effects of moderate-intensity resistance training and aerobic combined resistance training on obese college students. Meanwhile, based on subjective reports and objective measurement evidence, it revealed that aerobic combined resistance training has stronger application potential and is of reference value for formulating exercise intervention strategies for obese college students.

Firstly, we found that Baduanjin combined with resistance training and simple resistance training intervention has a significant improvement effect on the body composition of obese college students. This is consistent with previous studies, that is, Baduanjin intervention can significantly improve body composition indicators such as percent of body fat and BMI in obese people, and help reduce related diseases caused by obesity (Liu et al., 2018; Xie et al., 2023). Meanwhile, intermittent intervention of moderate and medium-to-high intensity physical exercise has a good effect on improving the body composition of obese female college students (Liu et al., 2023). The latest review literature points out that resistance exercise is an effective and safe method to improve the body composition of overweight and obese people (Dai et al., 2024). It can increase the basal metabolic rate of obese adolescents, improve the fat metabolism rate, and achieve the effect of reducing body fat (Monteiro et al., 2015). On this basis, this study found through different comparisons that Baduanjin combined with resistance training is more conducive to reducing the body fat percentage of obese college students than single resistance training, and the latter is more conducive to increasing muscle mass than the former. Related studies have shown that aerobic exercise is more effective in reducing fat (Prado et al., 2015), while resistance exercise mainly improves the body’s glucose and lipid metabolism by increasing muscle mass (Chen et al., 2019). 12 weeks of aerobic exercise and aerobic exercise combined with resistance training can improve the body composition (such as BMI, FM, and body fat percentage) of obese male college students, and the latter is better than the former (Zhao and Liang, 2019). Ho et al. (2012) found that after 12 weeks of aerobic exercise combined with resistance training, the weight and body fat mass reduction in overweight and obese adults was significantly higher than that in those who did aerobic exercise alone or resistance training alone. This can be explained by several potential mechanisms. For example, compared to single-mode aerobic or resistance training alone, combined aerobic and resistance training demonstrates superior efficacy in enhancing muscle protein synthesis (Colleluori et al., 2019). Moreover, this combined training modality has demonstrated enhanced efficacy in improving microcirculatory function among obese college students, including beneficial effects on microvascular reactivity, insulin resistance, and vascular endothelial cytokine levels (Xiao et al., 2022). These improvements subsequently contribute to more favorable body composition in obese individuals through multiple pathways such as increased energy expenditure and enhanced muscle mass. Furthermore, a study suggested that aerobic exercise yields better outcomes in short-term obesity management, whereas resistance training demonstrates more pronounced effects in long-term interventions, and it is recommended that exercise interventions for obese patients adopt a combined approach prioritizing aerobic exercise supplemented with resistance training (Chen et al., 2019).

Secondly, this study found that both types of exercise intervention have significant regulatory benefits on the autonomic nervous system of obese college students, showing that both interventions can significantly promote the improvement of SDNN, RMSSD, and HFnorm, and significantly reduce LFnorm and LF/HF. In other words, both exercise interventions can increase the vagus nerve activity of obese college students and inhibit the overactivity of the sympathetic nerve, thereby increasing the dynamic balance of the autonomic nerves. Heart rate variability (HRV) is an effective non-invasive indicator for monitoring and evaluating cardiac autonomic nervous activity (deAndrade et al., 2012). It quantifies the changes in heart rate during the R-R interval or each cardiac cycle, thereby reflecting autonomic nervous activity and quantitatively evaluating the cardiac sympathetic and vagal tone and balance (Malliani et al., 1991; Dreifus et al., 1993). Within a certain range, the greater the HRV fluctuation, the better the adaptability of the human autonomic nervous system and cardiovascular system to the external environment. Conversely, it means that the autonomic nervous system lacks a certain ability to respond to changes in the external environment (Tha et al., 2000). Studies have shown that obese people usually have relatively low levels of SDNN, RMSSD, and other indicators, and have vagus nerve and sympathetic nerve disorders (Wang and Liu, 2011). For example, there is a direct correlation between HRV activity in obese children and the body’s lipid metabolism and adipose cytokine content. Excessive obesity is an important cause of abnormal heart function in children (Zhang et al., 2017). Interestingly, different forms of physical exercise intervention can effectively improve the HRV of obese people, thereby improving the autonomic regulation function of the heart (Hynynen et al., 2010). Guo (Guo, 2015) found that long-term Tai Chi exercise can improve the overall regulatory function of the individual’s autonomic nervous system, which is manifested in a significant increase in the regulatory effect of the vagus nerve and a weakening of the sympathetic nervous system’s regulatory effect. It is worth noting that we also found that compared with resistance training alone, Baduanjin combined with resistance training has better regulatory benefits on the frequency domain indicators (LFnorm, HFnorm, and LF/HF) of obese college students, as shown in LFnorm and LF/HF have lower levels, while HFnorm has higher levels. Hynynen et al. (Hynynen et al., 2010) pointed out that aerobic exercise can increase vagal nerve tension and induce adaptive changes in HRV. At the same time, aerobic exercise can fully restore vagal nerve tension (Wang and Liu, 2011), and the improvement of cardiovascular function in obese male college students by aerobic exercise combined with resistance training may be related to the regulation of the balance of sympathetic-vagal nerve tension in obese college students by this exercise (Zhao and Liang, 2019). Therefore, we speculate that Baduanjin, as an aerobic exercise, is more conducive to improving the activity of HRV in obese college students and maintaining the dynamic balance of sympathetic/vagal nerves when combined with resistance training.

Moreover, we also explored the effects of two types of exercise intervention on blood lipid metabolism in obese college students. The results showed that after the intervention, TC, TG, and LDL-C in the two exercise groups showed a significant downward trend, and HDL-C showed a significant upward trend, while there was no significant change in the control group before and after the intervention. This is consistent with previous studies. For example, long-term Baduanjin exercise can effectively reduce the TG, TC, LDL-C levels, and atherosclerosis index of patients with hyperlipidemia, increase HDL-C levels, and improve blood lipid and lipoprotein metabolism, thereby effectively preventing and treating hyperlipidemia and reducing the risk of cardiovascular disease (Miao et al., 2009). Meanwhile, Baduanjin exercise can significantly improve the blood lipid metabolism and antioxidant stress levels of middle-aged and elderly people, which has a positive significance in preventing and delaying atherosclerosis (Zhang et al., 2016). Both aerobic exercise (Chen et al., 2019; Chen et al., 2020) and resistance training (Lira et al., 2010) can improve individual blood lipid metabolism. Among them, aerobic exercise primarily works by enhancing lipoprotein lipase activity, promoting triglyceride hydrolysis, and improving lipid metabolism levels (Lin et al., 2023), while resistance exercise mainly improves lipid metabolism indirectly by increasing muscle mass and basal metabolic rate (Santos et al., 2024). Further studies have shown that 12 weeks of aerobic exercise and aerobic exercise combined with resistance training can improve the cardiovascular function, total circulatory resistance, stroke volume, and cardiac output of obese male college students, and the latter is better than the former (Zhao and Liang, 2019). A 6-week aerobic exercise combined with resistance training intervention can effectively reduce weight, body fat percentage, insulin resistance and improve glucose and lipid metabolism in obese adolescents, and improve their endothelial function (Li et al., 2013). The results indicate that incorporating aerobic exercise into resistance training may be more conducive to promoting blood circulation and vascular endothelial function, effectively preventing lipid deposition in the vascular walls, while also increasing basal metabolic rate and energy expenditure, thereby synergistically improving blood lipid metabolism levels. In addition, there are certain differences in the exercise intensity required for different forms of exercise. For example, moderate-intensity resistance training is more effective in improving blood lipid metabolism indicators such as TG and HDL-C (Lira et al., 2010). Intermittent intervention of moderate and medium-to-high intensity physical exercise (such as brisk walking and skipping rope) has been shown to have a good effect on improving the blood lipid metabolism of obese female college students (Liu et al., 2023). There are also studies showing that after high-intensity interval training intervention, TC, TG, and LDL-C in obese college students (Lu and Xie, 2021) and people with dyslipidemia (He and Wang, 2019) showed a significant downward trend, while HDL-C showed an upward trend. In summary, physical exercise has a positive effect on improving blood lipid metabolism in obese people, but different forms of exercise may differ in the selection of exercise intensity. We believe that moderate-intensity aerobic combined with resistance training may be more in line with the physiological characteristics of obese people, and it is also easier to improve the compliance of participants and the intervention effect.

Finally, we also found that after the 8-week intervention, the subjective exercise experience level of the BRTG was significantly better than that of the RTG and CG. Additionally, the dropout rate of the BRTG was relatively lower compared to the RTG, indicating that the combined group may have generated more positive wellbeing and less psychological distress and fatigue during the exercise intervention. A study has shown that Baduanjin is characterized by its simplicity and ease of learning, as well as its gentle and slow nature, so individuals will not experience excessive discomfort during practice, and they will feel light and comfortable after exercising, and it is easier to persist and has a higher compliance rate than resistance exercises (Li et al., 2019). Based on this, it can be inferred that for obese college students, combining aerobic exercises such as Baduanjin based on resistance training can effectively improve their exercise experience and alleviate the adverse experiences such as muscle soreness and lactic acid accumulation caused by resistance training. This is relatively consistent with previous studies, for example, in patients with coronary heart disease, aerobics combined with resistance training is more effective in improving exercise tolerance and reducing skeletal muscle fatigue (Gayda et al., 2009), and also has a better effect on improving an individual’s fatigue perception and exercise tolerance (Grazioli et al., 2019). The results suggest that compared with single resistance training, aerobic exercise (such as Baduanjin) combined with resistance training seems to be more conducive to increasing the subjective exercise experience of obese college students, which provides a basis for improving their compliance.

However, this study also has some limitations: 1) As this is a preliminary study, the included physiological indicators related to obesity are limited, and whether there is a correlation or causal relationship between different indicators after exercise intervention needs to be verified in subsequent studies. 2) Both the Baduanjin and resistance training in this study were moderate intensities, and the dose-benefit relationship between different exercise intensities was not examined. Compared with low-intensity and high-intensity exercise interventions, it is still unknown whether moderate-intensity Baduanjin combined with resistance training is the best choice for health promotion weight loss, and lipid reduction in obese people. 3) In terms of study design, the relatively small sample size in this study, combined with the limited intervention duration, has somewhat constrained the scientific robustness of the findings. Future research could extend the intervention period to better reveal trends of change and increase the sample size to examine gender differences. 4) Participants’ daily physical activity levels were not objectively monitored in this study. Excessive additional physical activity may lead to deviations in body composition indicators, so future studies could utilize triaxial accelerometers to objectively measure participants’ daily physical activity levels.

## 5 Conclusion

Both resistance training alone and Baduanjin combined with resistance training have varying degrees of positive effects on improving body composition, autonomic nervous system activity, and blood lipid metabolism in obese college students. In comparison, the Baduanjin combined with resistance training is more conducive to reducing the body fat percentage and sympathetic nerve activity of obese individuals, enhancing the activity of the vagus nerve and the balance of the sympathetic/vagus nerve, and increasing the subjective exercise experience and compliance of participants. In contrast, single resistance training is more beneficial for improving the muscle mass of obese individuals. Overall, moderate-intensity Baduanjin combined with resistance training holds greater application potential for health promotion among obese college students and may represent a more suitable exercise regimen for this population.

## Data Availability

The original contributions presented in the study are included in the article/supplementary material, further inquiries can be directed to the corresponding author.
